# Investigating causal associations between pneumonia and lung cancer using a bidirectional mendelian randomization framework

**DOI:** 10.1186/s12885-024-12147-3

**Published:** 2024-06-11

**Authors:** Lujia Song, Dongsheng Wu, Jiayang Wu, Jiexi Zhang, Weimin Li, Chengdi Wang

**Affiliations:** 1 Department of Pulmonary and Critical Care Medicine, State Key Laboratory of Respiratory Health and Multimorbidity, Targeted Tracer Research and Development Laboratory, Med-X Center for Manufacturing, Frontiers Science Center for Disease-related Molecular Network, West China Hospital, Sichuan University, Chengdu, Sichuan China; 2 Department of Thoracic Surgery, Institute of Thoracic Oncology, West China Hospital, Sichuan University, Chengdu, Sichuan China; 3 Chengdu Medical College, Chengdu, Sichuan China

**Keywords:** Pneumonia, Lung cancer, Mendelian randomization, Causal relationship

## Abstract

**Background:**

Pneumonia and lung cancer are both major respiratory diseases, and observational studies have explored the association between their susceptibility. However, due to the presence of potential confounders and reverse causality, the comprehensive causal relationships between pneumonia and lung cancer require further exploration.

**Methods:**

Genome-wide association study (GWAS) summary-level data were obtained from the hitherto latest FinnGen database, COVID-19 Host Genetics Initiative resource, and International Lung Cancer Consortium. We implemented a bidirectional Mendelian randomization (MR) framework to evaluate the causal relationships between several specific types of pneumonia and lung cancer. The causal estimates were mainly calculated by inverse-variance weighted (IVW) approach. Additionally, sensitivity analyses were also conducted to validate the robustness of the causalty.

**Results:**

In the MR analyses, overall pneumonia demonstrated a suggestive but modest association with overall lung cancer risk (Odds ratio [OR]: 1.21, 95% confidence interval [CI]: 1.01 − 1.44, *P* = 0.037). The correlations between specific pneumonia types and overall lung cancer were not as significant, including bacterial pneumonia (OR: 1.07, 95% CI: 0.91 − 1.26, *P* = 0.386), viral pneumonia (OR: 1.00, 95% CI: 0.95 − 1.06, *P* = 0.891), asthma-related pneumonia (OR: 1.18, 95% CI: 0.92 − 1.52, *P* = 0.181), and COVID-19 (OR: 1.01, 95% CI: 0.78 − 1.30, *P* = 0.952). Reversely, with lung cancer as the exposure, we observed that overall lung cancer had statistically crucial associations with bacterial pneumonia (OR: 1.08, 95% CI: 1.03 − 1.13, *P* = 0.001) and viral pneumonia (OR: 1.09, 95% CI: 1.01 − 1.19, *P* = 0.037). Sensitivity analysis also confirmed the robustness of these findings.

**Conclusion:**

This study has presented a systematic investigation into the causal relationships between pneumonia and lung cancer subtypes. Further prospective study is warranted to verify these findings.

**Supplementary Information:**

The online version contains supplementary material available at 10.1186/s12885-024-12147-3.

## Background

Pneumonia is prevalent and often underestimated regarding the dreadful result [1], posing a significant threat to human health due to its high incidence. Pneumonia can arise from numerous different causes, predominantly bacteria and viruses [2]. Other non-infectious factors, like asthma, have also been highly linked to an increased risk of pneumonia [3]. While most patients recover, pneumonia has been associated with longer-term effects, including cardiovascular disease [4], cognitive decline [5], and impaired immunity [6]. Nevertheless, the relationship between pneumonia and lung cancer risk has not been comprehensively investigated.

Lung cancer accounts for the leading cause of cancer-related deaths worldwide [7]. Among the numerous cases, lung cancer exhibits considerable heterogeneity. Regarding the causal relationship between pneumonia and lung cancer susceptibility, a case–control study has reported that pneumonia elevated the risk of lung cancer with an odds ratio (OR) of up to 2.4 [8]. Similarly, another retrospective analysis suggested that pneumonia was significantly associated with an elevated 1-year incidence of lung cancer, suggesting the demand of follow-up imaging after pneumonia to rule out occult malignancies [9]. In a Danish nationwide study, patients who had experienced pneumonia were found to confront an eight-fold higher risk of developing lung cancer [10]. Conversely, there has also been conflicting point, underscoring a low incidence of lung cancer after pneumonia [11]. Given the contradictory evidence, the detailed relationship between pneumonia and the risk of lung cancer requires further exploration.

Genome-wide association studies (GWAS) have yielded myriad single nucleotide polymorphisms (SNPs) associations with traits. Mendelian randomization (MR) is a reliable method based on GWAS summary-level data to look into the causal relationships between exposure and outcome [12]. In the MR analysis, genetic variations can be leveraged as instrumental variables (IVs) to represent the specific exposures, which can largely avoid potential confounding factors and reverse causal effects [13]. Thus, compared to traditional observational designs, MR may provide more conclusive evidence regarding the causal relationships between pneumonia and lung cancer susceptibility. For example, a previous study has demonstrated that Corona Virus Disease 2019 (COVID-19) would have no significant causal relationship with the risk of lung cancer using MR [14]. Moreover, GWAS data also present opportunities to uncover shared genetic correlations across phenotypes, thus providing novel etiological perspectives [15, 16].

In this manuscript, we sought to assess the causal relationships between pneumonia and lung cancer with large-size GWAS data using MR approach. To avoid reverse causation, a bidirectional design was employed to investigate the relationship of lung cancer on pneumonia risk. In summary, this study has presented a systematic investigation into the causal relationships between pneumonia and lung cancer subtypes.

## Methods

### Prepositions of MR design

**Fig. 1:**
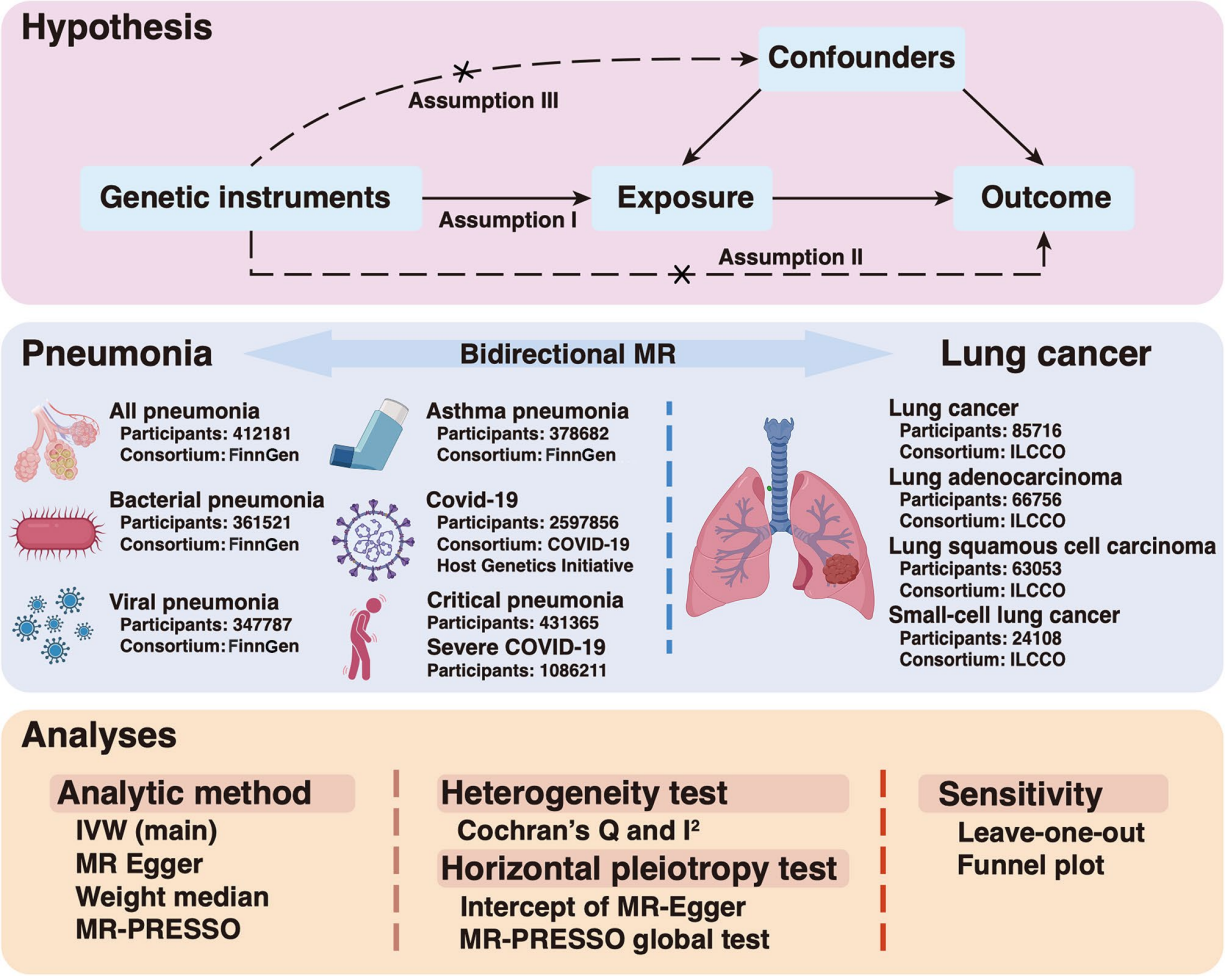
Schematic illustration illustrated Mendelian randomization assumptions. The assumptions included: (I) strong association between genetic variants and the chosen exposure; (II) no independent effect of genetic variants on the specific outcome; and (III) no association between genetic variants and potential confounders. MR: Mendelian randomization; COVID-19: Corona Virus Disease 2019; ILCCO: International Lung Cancer Consortium; IVW: Inverse-variance weighted; MR-PRESSO: Mendelian randomization-pleiotropy residual sum and outlier. Created with BioRender.com

In the bidirectional two-sample MR analyses, we adopted SNPs as IVs based on GWAS summary statistics from publicly available databases and recently published meta-analyses of GWAS data. To reduce the overlap in research population of exposure and outcome, we obtained GWAS datasets from two distinct European ancestry cohorts. To ensure valid causal estimates in MR analysis, three assumptions should be met: (I) strong association between genetic variants and exposure; (II) no independent effect of genetic variants on the outcome; and (III) no association between genetic variants and confounders (Fig. 1). Our study was based upon publicly released data, and all research databases listed here had received an ethics approval.

### Data sources for multiple types of pneumonia and lung cancer

Detailed information about the features of every dataset incorporated in this study is provided specifically in Table 1. We retrieved summary-level GWAS data for pneumonia from the hitherto latest FinnGen database (www.finbb.fi). The FinnGen database is a large-scale, population-based biobank study conducted in Finland, aiming to represent a comprehensive genomic dataset and phenotypic information for over 500,000 European participants [17]. Based on different pathogenesis of pneumonia, pneumonia phonotypes comprising all pneumonia (63,377 cases and 348,804 controls), bacterial pneumonia (17,511 cases and 344,010 controls), viral pneumonia (3,777 cases and 344,010 controls), and asthma − related pneumonia (13,185 cases and 365,497 controls) were retrieved. Moreover, summary data of the largest GWAS on COVID-19 (122,616 cases and 2,475,240 controls) as well as severe COVID-19 (13,769 severe cases and 1,072,442 controls) were obtained from the COVID-19 Host Genetics Initiative and the European ancestry resources were selected [18]. Moreover, we also accessed GWAS data of critical pneumonia from 431,365 European individuals, including 2,758 cases and 428,607 controls [19].

**Table 1:** Summary of data source of different traits

Traits		Case/Control^#^	Population	Year	Consortium	ID
Pneumonia types	All pneumonia	63,377/348,804	European	2023	FinnGen	www.finngen.fi/en
	Bacterial pneumonia	17,511/344,010	European	2023	FinnGen	www.finngen.fi/en
	Viral pneumonia	3,777/344,010	European	2023	FinnGen	www.finngen.fi/en
	Asthma-related pneumonia	13,185/365,497	European	2023	FinnGen	www.finngen.fi/en
	COVID-19	122,616/2,475,240	European	2020	COVID-19 Host Genetics Initiative	PMID: 32404885
Pneumonia severity	Critical pneumonia	2,758/428,607	European	2023	Not applicable	PMID: 36716318
	COVID-19 severe illness	13,769/1,072,442	European	2020	COVID-19 Host Genetics Initiative	PMID: 32404885
Lung cancer types	Lung cancer	29,266/56,450	European	2022	International Lung Cancer Consortium	PMID: 28604730
	Lung adenocarcinoma	11,273/55,483	European	2022	International Lung Cancer Consortium	PMID: 28604730
	Lung squamous cell carcinoma	7,426/55,627	European	2022	International Lung Cancer Consortium	PMID: 28604730
	Small-cell lung cancer	2,664/21,444	European	2022	International Lung Cancer Consortium	PMID: 28604730
Confounding risk factors	Body mass index	461,460	European	2018	UK biobank	ukb-b-19953
	Cigarettes smoked per day	337,334	European	2019	GWAS & Sequencing Consortium of Alcohol and Nicotine use	PMID: 30643251
	Smoking: ever vs. current	407,766/139,453	European	2019	GWAS & Sequencing Consortium of Alcohol and Nicotine use	PMID: 30643251
	Smoking: ever vs. never	557,337/674,754	European	2019	GWAS & Sequencing Consortium of Alcohol and Nicotine use	PMID: 30643251
	Drinking per week	941,280	European	2019	GWAS & Sequencing Consortium of Alcohol and Nicotine use	PMID: 30643251

*Note* ^#^sample size of categorical variables was presented as case/control, while sample size of continuous variables was presented as total sample size

The GWAS data for lung cancer were retrieved from an aggregated analysis conducted by the International Lung Cancer Consortium (ILCCO) [20], a global collaboration of researchers focused on lung cancer. This GWAS data of lung cancer comprised 29,266 cases and 56,450 controls in total. Stratified by histologic subtypes, there were lung adenocarcinoma (LUAD) (11,273 cases and 55,483 controls), lung squamous cell carcinoma (LUSC) (7,426 cases and 55,627 controls), and small-cell lung cancer (SCLC) (2,664 cases and 21,444 controls).

### Data sources for potential risk factors

To assess the causal relationship between pneumonia and potential risk factors, we performed further inverse-variance weighted (IVW) analyses to investigate whether pneumonia potentially affected lung cancer risk through these factors. In this section, risk factors associated with risk of cancer commonly considered in the MR analysis were included, namely body mass index (BMI), smoking status (cigarettes smoked per day, ever vs. current smoker, and ever vs. never smoker), and alcohol consumption. Regarding smoking status and alcohol consumption, GWAS data were downloaded from the GWAS & Sequencing Consortium of Alcohol and Nicotine use (GSCAN) [21]. The GWAS data for BMI were retrieved from the MRC-IEU OpenGWAS database (https://gwas.mrcieu.ac.uk/). Table 1 depicts the detailed information of GWAS summary data.

### Selection of SNPs

To ensure the appropriate selection of SNPs as IVs for our study, several criteria were applied. Firstly, to obtained more genetic instruments, the threshold for single SNP was set as *P* < 5 × 10^− 6^. Secondly, regarding the clumping process, a linkage disequilibrium (LD) algorithm was employed to maintain independence among the SNPs (r^2^ = 0.001 and window size = 10 Mb) [22]. Thirdly, we checked every SNP via Phenoscanner and GWAS catalog [23, 24]. In this way, we could adequately assess whether these SNPs were associated with potential confounders at the genome-wide significance threshold of *P* < 5 × 10^–8^. SNPs associated with smoking status, body mass index, alcohol intake, and any malignancy for IVs of pneumonia were removed, and the remaining SNPs were utilized as the IVs in the subsequent MR analyses.

### Estimation of causal association

Prior to conducting the analysis, we performed data harmonization to align the effect alleles of the exposure and outcome variables to the forward strand. This alignment was carried out based on specified information or inferred from allele frequencies. Additionally, palindromic genetic variants were excluded from further MR analyses [25]. For causal estimate, we employed the IVW, MR Egger, and weighted median methods. IVW approach could combine SNP-specific ratio estimates, regressing the coefficient of outcome against that of the exposure without the intercept term [26]. If no heterogeneity was detected, the fixed-effect IVW method was performed. In cases where heterogeneity was detected, the multiplicative random effects IVW approach was employed. Different from the IVW method, MR-Egger allows for detecting and correcting for the potential bias caused by the presence of directional pleiotropy [27]. The weighted median method could maintain robustness against the influence of invalid instruments, accommodating up to half of invalid SNPs [28]. To further test the robustness of the causal estimates, sensitivity analyses were thus conducted containing the heterogeneity test measured by Cochran’s Q statistic, pleiotropy by MR-Egger intercept test and MR pleiotropy residual sum and outlier (MR-PRESSO). Additionally, to evaluate the potential impact of each SNP on the IVW estimate, leave-one-out analyses were performed, which removed one SNP at a time. Funnel plots were utilized to illustrate the selection bias of IVs. A prespecified significance threshold of *P* < 1.25 × 10^− 3^ (adjusted for multiple testing: *P* = 0.05/40, considering 5 pneumonia types and 4 lung cancer types in a bidirectional design was applied using the Bonferroni correction in the bidirectional MR analyses. All statistical analyses were performed in R software (version 4.2.2) using the R packages “TwoSampleMR” (version 1.0) and “MRPRESSO” (version 0.5.6) [29, 30].

### Statistical power and F‑statistics

Based on an online calculator (https://shiny.cnsgenomics.com/mRnd/) [31], the power in our MR analyses were calculated. The calculation incorporated the type I error of 0.05, proportion of cases (Table 1), explained genetic variation (R^2^) (Supplementary Table 1), and OR from IVW analyses (Supplementary Table 3). R^2^ of each SNP was equal to 2×EAF×(1 − EAF)×β^2^, where EAF represented the effect allele frequency, while β denoted the estimated genetic effect on the exposure [32]. The F statistic in MR analysis measured instrument strength based on R^2^, sample size (N), as well as the number of instruments (K), which could be calculated by: $\text{F}=\left(\frac{\text{N}-1-\text{K}}{\text{K}}\right)\left(\frac{{\text{R}}^{2}}{1-{\text{R}}^{2}}\right)$[33]. Mitigating weak instrument bias is paramount in the design and analysis of MR analysis, and an F statistic exceeding 10 could indicate a sufficient strength [34].

### Genetic correlation analysis

To understand the potential shared genetic basis between pneumonia and lung cancer, we conducted a genome-wide genetic correlation analysis. This method involves utilizing large-scale genomic data from GWAS to calculate the genome-wide genetic correlations (r_g_) between different trait pairs [15]. These correlations quantify the average shared genetic influences between traits, independent of environmental factors. We employed the Linkage Disequilibrium Score Regression (LDSC) method, a robust statistical algorithm that calculates genetic correlations by regressing the product of z-scores for two traits on the LD patterns across SNPs spanning the human genome [16]. In brief, this analysis will suggest the genetic architecture underlying the relationships between pneumonia and lung cancer.

## Results

Details of SNPs with pneumonia as the exposure are presented in Supplementary Table 1. The selected SNPs could explain 1.57%, 2.80%, 10.25%, 9.86%, and 1.75% of the variance in overall pneumonia, bacterial pneumonia, viral pneumonia, asthma-related pneumonia, and COVID-19, respectively. And F-statistics were all above 10, which suggested a sufficient strength. The statistical powers of MR results are presented in Supplementary Table 2.

**Fig. 2:**
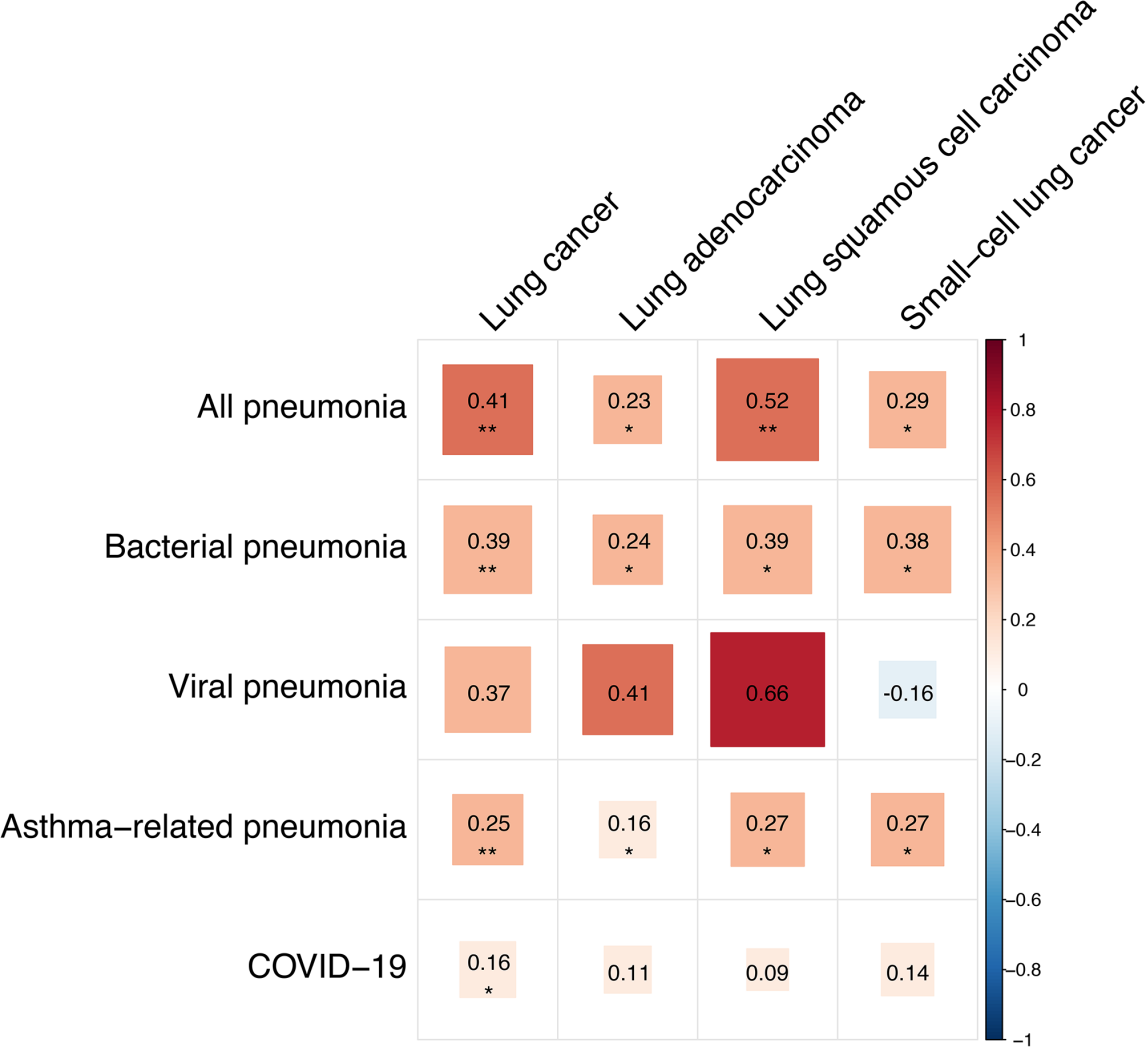
Genetic correlations (r_g_) estimated between pneumonia and lung cancer using genome-wide SNPs via LDSC method. The pairwise estimate was reported in each case, with asterisks *denoting statistical significance at a *P*-value threshold of 0.05, and asterisks **denoting Bonferroni-corrected significance at a *P*-value threshold of 0.05/20. The colors of the box indicated the magnitude of correlation. SNP: Single nucleotide polymorphism; LDSC: Linkage disequilibrium score regression; COVID-19: Corona Virus Disease 2019

The genetic correlation analysis demonstrated an intrinsic genome-wide sharing between pneumonia and lung cancer. As shown in Fig. 2, the genetic correlations were pronounced between these two major diseases. For example, there was evidence on significant shared genetic basis between all pneumonia with overall lung cancer (r_g_=0.41), LUAD (r_g_=0.23), LUSC (r_g_=0.52) and SCLC (r_g_=0.29). Inspired by these findings, we systematically analyzed the causal relationships between pneumonia and lung cancer.

### General effect of pneumonia on lung cancer

**Fig. 3:**
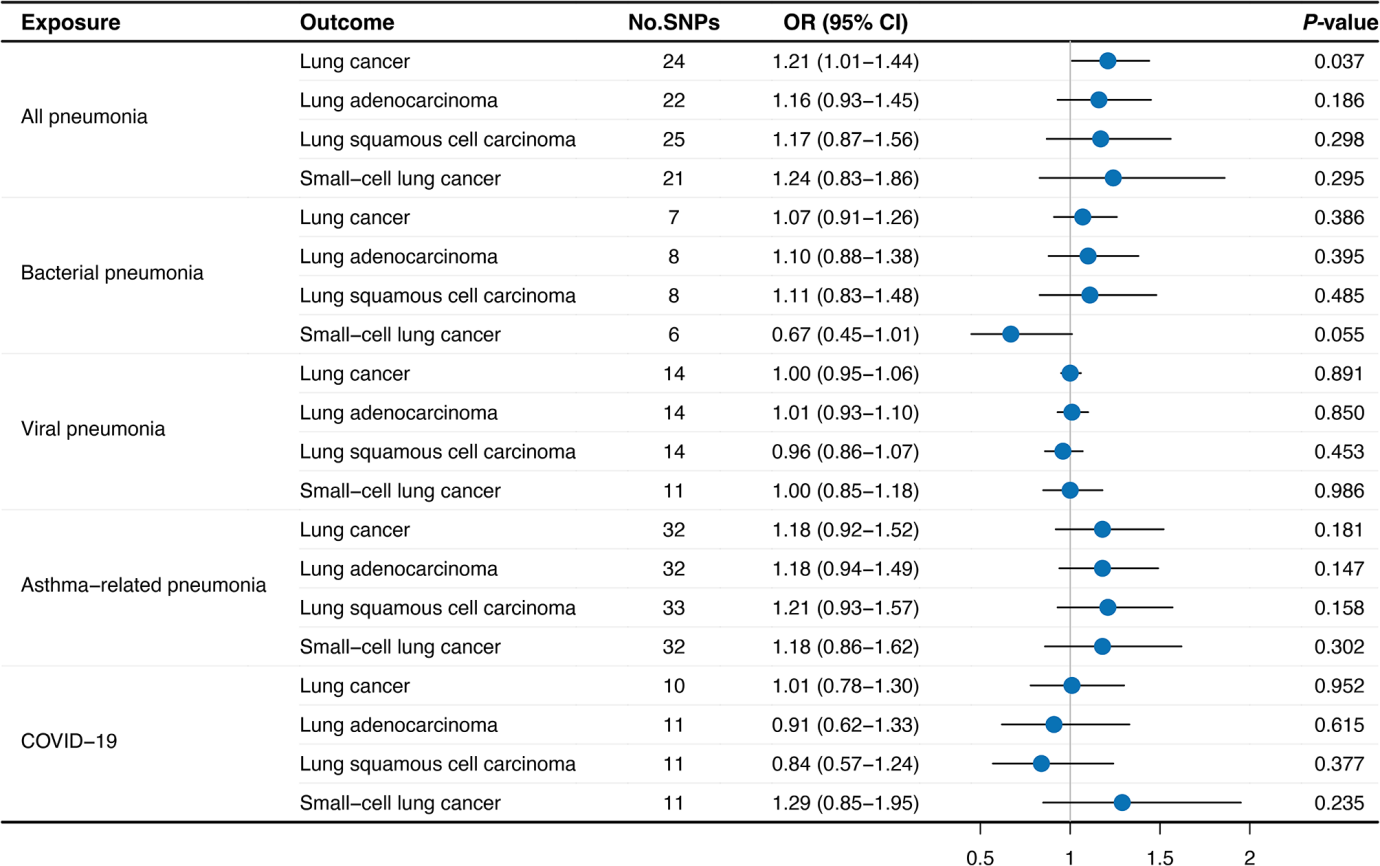
Mendelian randomization using IVW method estimated the causal effects of pneumonia on lung cancer susceptibility. IVW: Inverse-variance weighted; SNP: Single nucleotide polymorphism; OR: Odds ratio; CI: Confidence interval; COVID-19: Corona Virus Disease 2019

Figure 3 has illustrated a comprehensive landscape of IVW estimates when pneumonia served as the exposure. We found a modest yet potentially causal relationship between overall pneumonia and overall lung cancer (Odds ratio [OR]: 1.21, 95% confidence interval [CI]: 1.01 − 1.44, *P* = 0.037). This association was supported by a high statistical power of 93% (Supplementary Table 2). However, the correlations between specific pneumonia subtypes and overall lung cancer were not as pronounced. Bacterial pneumonia (OR: 1.07, 95% CI: 0.91–1.26, *P* = 0.386), viral pneumonia (OR: 1.00, 95% CI: 0.95–1.06, *P* = 0.891), asthma-related pneumonia (OR: 1.18, 95% CI: 0.92–1.52, *P* = 0.181), and COVID-19 pneumonia (OR: 1.01, 95% CI: 0.78–1.30, *P* = 0.952) did not exhibit evident associations with lung cancer, as well as other lung cancer subtypes (Fig. 3). Meanwhile, the weighted median along with MR-Egger regression approaches demonstrated similar trends (Supplementary Table 3). To supplement the primary analyses, we also conducted additional MR analyses utilizing IVW approach to evaluate the potential causal effect of pneumonia on the potential confounding risk factors of lung cancer, including BMI, smoking status, and drinking status (Supplementary Table 4). And no causal relationships could be found between most pneumonia types and these risk factors, except for a marginal correlation between COVID-19 and drinking status (OR: 1.03, 95% CI: 1.00–1.06, *P* = 0.038). Therefore, we believe that our findings could indicate a potential causal relationship of pneumonia with increased lung cancer susceptibility, but considering the significant but modest association, further validations will be needed.

To add to the clinical relevance of this study, we also analyzed the association between severe pneumonia types and lung cancer. Based on GWAS data availability, we looked into specific traits including critical pneumonia and very severe respiratory confirmed COVID-19. The result showed that critical pneumonia (OR: 1.00, 95% CI: 0.98–1.02, *P* = 0.841) and severe COVID-19 (OR: 1.02, 95% CI: 0.96–1.07, *P* = 0.577) had no direct causal effect on increased lung cancer risks (Supplementary Table 5).

### Sensitivity analysis

In the sensitivity analysis, we adopted Cochran’s Q test to detect heterogeneity (Supplementary Table 6). Because we had leveraged the random-effects IVW MR approach in cases we detected heterogeneity, our results still remained applicable. In our study, different methods including IVW, weighted median, and MR-Egger showed consistent estimates, indicating robustness of these findings. Furthermore, the intercepts evaluated via MR-Egger did not exhibit statistically significant *P*-values (Supplementary Table 7), suggesting our results were not impacted by pleiotropy. Additionally, the leave-one-out analyses barely detect SNPs that might possibly cast a substantial influence on the final estimates, and the funnel plots did not reveal significant evidence of bias in evaluating potential biases in the genetic IVs (Supplementary Figs. 1–3).

### Bidirectional analyses showing the causal effect of lung cancer on pneumonia

**Fig. 4:**
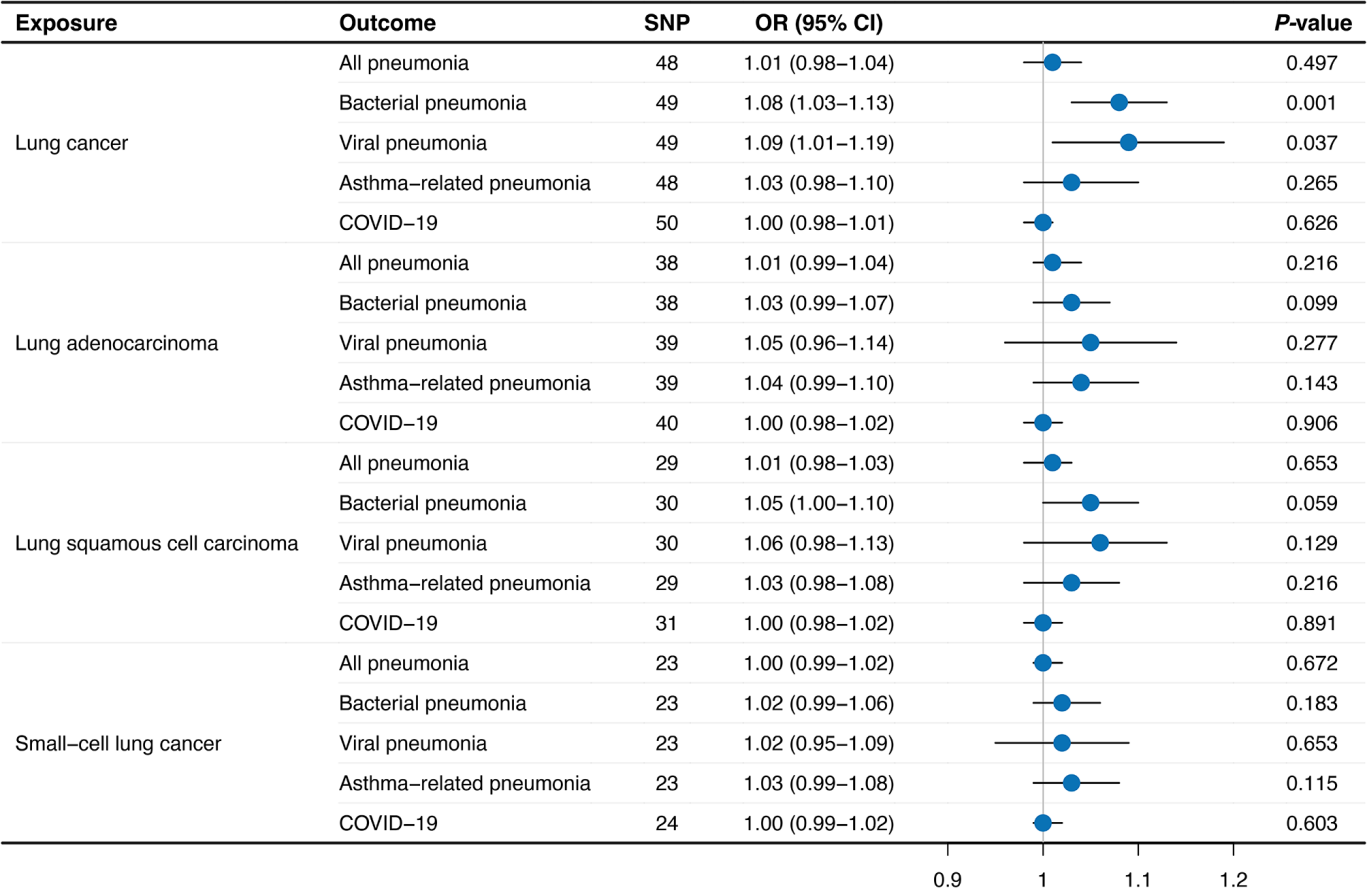
Mendelian randomization using IVW method estimated the causal effects of lung cancer on pneumonia risk. IVW: Inverse-variance weighted; SNP: Single nucleotide polymorphism; OR: Odds ratio; CI: Confidence interval; COVID-19: Corona Virus Disease 2019

On the other hand, we further investigated the causal relationship of lung cancer on pneumonia (Fig. 4). Supplementary Table 8 provides details of the specific SNPs utilized in this section. When lung cancer was considered the exposure, we uncovered a crucial causal link between overall lung cancer and elevated risks of bacterial pneumonia (OR: 1.08, 95% CI: 1.03–1.13, *P* = 0.001) and viral pneumonia (OR: 1.09, 95% CI: 1.01–1.19, *P* = 0.037). Moreover, the findings suggested that diverse types of lung cancer exhibited a modest but stable tendency to be possibly associated with higher susceptibility of specific pneumonia types, although these associations were not as statistically evident.

## Discussion

Motivated by the significant genetic correlations between pneumonia and lung cancer, this study has been the first to systematically investigate the causal relationships between these two major respiratory diseases with two-sample MR approach, and our results were built upon the hitherto most recent genetic data. We included a wide range of pneumonia and lung cancer types, and found a significant but modest causal relationship of overall pneumonia on lung cancer susceptibility. Reversely, with lung cancer as the exposure, the risks of developing bacterial and viral pneumonia were specifically elevated in lung cancer patients.

We found a modest yet suggestive increase of lung cancer risks in pneumonia patients. This effect presented in the overall pneumonia and lung cancer collectively, while the relatively smaller size of the GWAS data on other traits might limit the statistical power, rendering less significant correlation in specific disease types. But supported by a high statistical power of 93%, the potential causal relationship between pneumonia and lung cancer susceptibility generally should be noticed. And this finding has added to the current evidence of the post-pneumonia effects, also consistent with most earlier observational studies which have reported higher lung cancer risk following pneumonia based on conventional regression approaches. For example, a previous study covering 22,034 patients with pneumococcal pneumonia and 88,136 controls found increased lung cancer risk after pneumococcus infection [35]. And another study also stressed a higher lung cancer incidence after pneumonia in smokers [9]. In addition, the time period during which pneumonia might influence lung cancer risk had been discussed. The SYNERGY project, which collected information on a variety of previous respiratory diseases based on case-control studies, domonstrated that within 2 years after pneumonia, the risks for lung cancer were increased, but the impact did not exist after the time period of 2 years [36]. And another nationwide large-scale retrospective study suggested an elevated incidence ratio of lung cancer diagnosis maintaining beyond 5 years months following pneumonia [10]. Alternatively, an existing study has contrarily proposed a very low incidence of lung cancer new cases after pneumonia [11], constituting a minor side of the discussion. Given the intrinsic limitations of observational design in the inference of causality, there has been continuous doubt raised on the confounding factors [37, 38]. Herein, in the current study, we adopted MR design and thus minimized the confounding effects. We found the modest positive correlation between pneumonia and lung cancer susceptibility, which still needs to be further investigated in the larger cohorts or in prospective studies.

Regarding the mechanisms underlying the potential higher risks of lung cancer with pneumonia serving as the exposure, the enduring post-pneumonia effects including permanent lung damage, dysregulated immune function, and extrapulmonary complications have previously been reported [39–41]. Maintaining lung homeostasis requires a balance between immune resistance and the resilience of tissue [40]. When pneumonia occur, the immune system functions to combat invading agents, which simultaneously could cause tissue damage [41]. Patients after COVID-19 pneumonia are left with impaired lungs, and the abnormal lung function might even last for long [39, 40]. Therefore, a proper management after pneumonia are still imperative to monitor other potential long-term effects including lung cancer.

In our study, we also suggested elevated risks of bacterial and viral pneumonia among patients with lung cancer. This heightened susceptibility is primarily attributable to compromised immune functions in lung cancer patients. Immune system, pivotal in the defense against pathogenic invasion, is notably disrupted by tumors throughout the body [42]. As elucidated through established analyses especially using single-cell technique, significant alterations in immune cell populations, including but not limited to suppressed T cell activity [43], aberrations in B cell functions [44], compromised dendritic cell functions [45], and accumulation of immunosuppressive neutrophils [46], have been reported in lung cancer patients. Bacterial pneumonia predominantly caused by pathogens such as Streptococcus species, and viral pneumonia caused by a wide variety of viruses like influenza viruses, present the two major pneumonia types as the major cause of incidence and mortality [2, 47], underscoring the critical nature of the risks. Herein, we suggest the pressing need for meticulous pneumonia prophylaxis in the clinical management of lung cancer patients, particularly for infection control measures within hospital settings, thereby mitigating additional health complications and enhancing overall patient outcomes.

These findings have been reliable with the exclusion of effects brought about by major confounding factors. We especially excluded SNPs related with smoking status and BMI. And in our additional MR analyses, evidence for a causal association of the pneumonia subtypes on the potential risk factors was minimized, thus rendering the independence of our study from confounding impacts. Indeed, smoking played a key role in both pneumonia and lung cancer susceptibility. On the one hand, the relationship between smoking and the susceptibility of pneumonia is evident. Inducing physical airway changes like cilia loss and mucus overproduction, smoking has long been found to be a crucial risk factor which could incur a 2- to 4-fold elevated risk of pneumonia [48, 49]. These effects might partially explained by the suppression of the immune system caused by tobacco smoking [50]. On the other hand, smoking accounts for the vast majority among lung cancer cases [51]. Given the enormous impact smoking might have in the long term, lung cancer screening is recommended for heavy smokers aged 55–74 years old with a 30 pack-year history of smoking who are more prone to harbor lung cancer [52]. As is also with BMI, body fatness has been linked to higher risk for multiple cancers including lung cancer [53], and could also impact the pneumonia risk in a dose-related manner [54]. Our MR strategy has eliminated the potential confounding caused by BMI as well.

Our proposed modest yet significant link between pneumonia and lung cancer risk will potentially serve as a reference in clinical practice to recommend screening for lung cancer in post-pneumonia status. As early detection is crucial in lung cancer management, the pursuit for sensitive and reliable features in either clinical assessment or molecular scale is still warranted. From the clinical perspective, respiratory conditions have the potential to cast an impact on lung cancer risks. For example, chronic obstructive pulmonary disease (COPD) has been widely acknowledged as a relative element associated with lung cancer regardless of democratic factors and smoking history [55, 56]. Generally, lung function could predict lung cancer risks, acting as a relevant indicator for lung cancer screening [57]. At the molecular level, high-throughput sequencing of circulating tumor DNA has emerged as effective in differentiating lung cancer from other benign lung nodules, empowering early-stage diagnosis [58, 59]. Blood proteins, DNA methylation features, and RNA airway signatures are all promising candidates for molecular biomarkers in detecting lung cancer at an earlier stage [60–62].

The current study had clear strengths. The foremost strength of our study was that the results generated by MR analyses were not influenced by classical types of confounding factors or reverse causation that might bias findings in other observational settings. And our results could potentially inform ongoing or future trials into the traits affecting lung cancer onset. However, there were several limitations to consider for our study. Firstly, our analyses were based on GWAS summary-level data, which limited our ability to investigate potential confounding factors or individual-level characteristics. And although we adopted a relatively large GWAS data, our sample sizes might still be limited to detect more modest causal associations between pneumonia and lung cancer. In addition, we focused on only a few common pneumonia and lung cancer subtypes. In the foreseeable future, continued research in larger cohorts and in-depth investigation into the underlying disease mechanisms are required to further understand the complicated relationships between pneumonia and lung cancer types.

## Conclusion

This bidirectional MR study demonstrated a suggestive but modest causal relationship of pneumonia on overall lung cancer, as well as a higher risk of developing bacterial and viral pneumonia in lung cancer patients. Further large-scale, prospective study is warranted to verify these findings.

### Electronic supplementary material

Below is the link to the electronic supplementary material.

Supplementary Material 1

Supplementary Material 2

## Data Availability

The summary-level data used in this study can be obtained from public datasets GWAS summary statistics for lung cancer are from https://www.ebi.ac.uk/gwas/publications/28604730. GWAS summary statistics for most pneumonia types can be consulted at https://finngen.gitbook.io/documentation/data-download (R10 release). GWAS summary statistics of the COVID-19 Host Genetics Initiative are accessible at https://www.covid19hg.org/. GWAS summary data for critical pneumonia are available from GWAS catalog (ID: GCST90281170). More details of the approaches as well as the codes are available at https://mrcieu.github.io/TwoSampleMR/.

## Abbreviations

BMI: Body mass index
CI: Confidence interval
COPD: Chronic obstructive pulmonary disease
COVID-19: Corona Virus Disease 2019
EAF: Effect allele frequency
GWAS: Genome-wide association study
GSCAN: GWAS & Sequencing Consortium of Alcohol and Nicotine use
ILCCO: International Lung Cancer Consortium
IV: Inverse-variance
IVW: Inverse variance weighted
LC: Lung cancer
LD: Linkage disequilibrium
LDSC: Linkage disequilibrium score regression
LUAD: Lung adenocarcinoma
LUSC: Lung squamous cell carcinoma
MR: Mendelian randomization
MR-PRESSO: Mendelian randomization-pleiotropy residual sum and outlier
OR: Odds ratio
SCLC: Small-cell lung cancer
SNP: Single nucleotide polymorphism

## References

[1] Wunderink RG, Waterer GW (2014). Clinical practice. Community-acquired pneumonia. N Engl J Med.

[2] Sattar SBA, Sharma S. Bacterial pneumonia. in StatPearls. 2024. 30020693

[3] Edwards MR, Bartlett NW, Hussell T, Openshaw P, Johnston SL (2012). The microbiology of asthma. Nat Rev Microbiol.

[4] Corrales-Medina VF, Musher DM, Shachkina S, Chirinos JA (2013). Acute pneumonia and the cardiovascular system. Lancet.

[5] Ceban F (2022). Fatigue and cognitive impairment in Post-COVID-19 syndrome: a systematic review and meta-analysis. Brain Behav Immun.

[6] Sapey E (2017). Pulmonary infections in the elderly lead to impaired neutrophil targeting, which is improved by Simvastatin. Am J Respir Crit Care Med.

[7] Sung H (2021). Global Cancer statistics 2020: GLOBOCAN estimates of incidence and mortality worldwide for 36 cancers in 185 countries. CA Cancer J Clin.

[8] Ramanakumar AV, Parent M-E, Menzies D, Siemiatycki J (2006). Risk of lung cancer following nonmalignant respiratory conditions: evidence from two case-control studies in Montreal, Canada. Lung Cancer.

[9] Shepshelovich D (2022). Incidence of lung cancer following pneumonia in smokers: a population-based study. QJM.

[10] Søgaard KK (2015). Pneumonia and the incidence of cancer: a Danish nationwide cohort study. J Intern Med.

[11] Tang KL, Eurich DT, Minhas-Sandhu JK, Marrie TJ, Majumdar SR (2011). Incidence, correlates, and chest radiographic yield of new lung cancer diagnosis in 3398 patients with pneumonia. Arch Intern Med.

[12] Burgess S, Scott RA, Timpson NJ, Davey Smith G, Thompson SG (2015). Using published data in mendelian randomization: a blueprint for efficient identification of causal risk factors. Eur J Epidemiol.

[13] Zhu Z, Hasegawa K, Camargo CA, Liang L (2021). Investigating asthma heterogeneity through shared and distinct genetics: insights from genome-wide cross-trait analysis. J Allergy Clin Immunol.

[14] Li J (2023). Causal effects of COVID-19 on cancer risk: a mendelian randomization study. J Med Virol.

[15] Bulik-Sullivan B (2015). An atlas of genetic correlations across human diseases and traits. Nat Genet.

[16] Bulik-Sullivan BK (2015). LD score regression distinguishes confounding from polygenicity in genome-wide association studies. Nat Genet.

[17] Kurki MI (2023). FinnGen provides genetic insights from a well-phenotyped isolated population. Nature.

[18] The COVID-19 (2020). Host Genetics Initiative, a global initiative to elucidate the role of host genetic factors in susceptibility and severity of the SARS-CoV-2 virus pandemic. Eur J Hum Genet.

[19] Hamilton FW (2023). Therapeutic potential of IL6R blockade for the treatment of sepsis and sepsis-related death: a mendelian randomisation study. PLoS Med.

[20] McKay JD (2017). Large-scale association analysis identifies new lung cancer susceptibility loci and heterogeneity in genetic susceptibility across histological subtypes. Nat Genet.

[21] Liu M (2019). Association studies of up to 1.2 million individuals yield new insights into the genetic etiology of tobacco and alcohol use. Nat Genet.

[22] Berisa T, Pickrell JK. Approximately independent linkage disequilibrium blocks in human populations. Bioinformatics. 2016;32(2):283–285. 10.1093/bioinformatics/btv546 PMC4731402 26395773

[23] Kamat MA (2019). PhenoScanner V2: an expanded tool for searching human genotype-phenotype associations. Bioinformatics.

[24] Buniello A (2019). The NHGRI-EBI GWAS catalog of published genome-wide association studies, targeted arrays and summary statistics 2019. Nucleic Acids Res.

[25] Hartwig FP, Davies NM, Hemani G, Davey Smith G (2016). Two-sample mendelian randomization: avoiding the downsides of a powerful, widely applicable but potentially fallible technique. Int J Epidemiol.

[26] Burgess S, Butterworth A, Thompson SG (2013). Mendelian randomization analysis with multiple genetic variants using summarized data. Genet Epidemiol.

[27] Bowden J, Davey Smith G, Burgess S (2015). Mendelian randomization with invalid instruments: effect estimation and bias detection through Egger regression. Int J Epidemiol.

[28] Bowden J, Davey Smith G, Haycock PC, Burgess S (2016). Consistent estimation in mendelian randomization with some Invalid instruments using a weighted median estimator. Genet Epidemiol.

[29] Hemani G et al. The MR-Base platform supports systematic causal inference across the human phenome. elife. 2018;7:e34408. 10.7554/eLife.34408 PMC5976434 29846171

[30] Verbanck M, Chen CY, Neale B, Do R (2018). Detection of widespread horizontal pleiotropy in causal relationships inferred from mendelian randomization between complex traits and diseases. Nat Genet.

[31] Brion MJ, Shakhbazov K, Visscher PM (2013). Calculating statistical power in mendelian randomization studies. Int J Epidemiol.

[32] Wu D (2023). Genetically predicted childhood body mass index and lung cancer susceptibility: A two-sample Mendelian randomization study. Cancer Med.

[33] Palmer TM (2012). Using multiple genetic variants as instrumental variables for modifiable risk factors. Stat Methods Med Res.

[34] Burgess S, Thompson SG (2011). Avoiding bias from weak instruments in Mendelian randomization studies. Int J Epidemiol.

[35] Lin TY (2014). Increased lung cancer risk among patients with pneumococcal pneumonia: a nationwide population-based cohort study. Lung.

[36] Denholm R (2014). Is previous respiratory disease a risk factor for lung cancer?. Am J Respir Crit Care Med.

[37] Chen CC, Lee YT, Wang PH, Chen SC. Occurrence of lung cancer after hospitalisation for pneumonia among smokers. QJM. 2023;116:958. 10.1093/qjmed/hcad131 37307075

[38] Chang R, Yeh WB, Lai CC (2023). Incidence of lung cancer following pneumonia in smokers: correspondence. QJM.

[39] Mo X, Jian W, Su Z, et al. Abnormal pulmonary function in COVID-19 patients at time of hospital discharge. Eur Respir J. 2020;55(6):2001217. 10.1183/13993003.01217-2020 PMC7236826 32381497

[40] Torres A (2021). Pneumonia. Nat Reviews Disease Primers.

[41] Quinton LJ, Walkey AJ, Mizgerd JP (2018). Integrative physiology of Pneumonia. Physiol Rev.

[42] Allen BM (2020). Systemic dysfunction and plasticity of the immune macroenvironment in cancer models. Nat Med.

[43] Herbst RS, Morgensztern D, Boshoff C (2018). The biology and management of non-small cell lung cancer. Nature.

[44] Chen J (2020). Single-cell transcriptome and antigen-immunoglobin analysis reveals the diversity of B cells in non-small cell lung cancer. Genome Biol.

[45] Wang JB, Huang X, Li FR (2019). Impaired dendritic cell functions in lung cancer: a review of recent advances and future perspectives. Cancer Commun (Lond).

[46] Koyama S (2016). STK11/LKB1 deficiency promotes neutrophil recruitment and proinflammatory cytokine production to suppress T-cell activity in the lung tumor microenvironment. Cancer Res.

[47] Freeman AM, Leigh. J.T.R. Viral pneumonia. in *StatPearls*. 2024. 30020658

[48] Arcavi L, Benowitz NL (2004). Cigarette smoking and infection. Arch Intern Med.

[49] Nuorti JP (2000). Cigarette smoking and invasive pneumococcal disease. Active bacterial core Surveillance Team. N Engl J Med.

[50] Sopori M (2002). Effects of cigarette smoke on the immune system. Nat Rev Immunol.

[51] Subramanian J, Govindan R (2007). Lung cancer in never smokers: a review. J Clin Oncol.

[52] Wood DE (2018). Lung Cancer Screening, Version 3.2018, NCCN Clinical Practice guidelines in Oncology. J Natl Compr Canc Netw.

[53] Lauby-Secretan B (2016). Body fatness and Cancer–viewpoint of the IARC Working Group. N Engl J Med.

[54] Nie W (2014). Obesity survival paradox in pneumonia: a meta-analysis. BMC Med.

[55] Young RP (2009). COPD prevalence is increased in lung cancer, independent of age, sex and smoking history. Eur Respir J.

[56] Cheng LL, et al. Clinical characteristics of tobacco smoke-induced versus biomass fuel-induced chronic obstructive pulmonary disease. J Transl Int Med. 2015;3:126–9. 10.1515/jtim-2015-0012 PMC4936465 27847900

[57] Calabrò E (2010). Lung function predicts lung cancer risk in smokers: a tool for targeting screening programmes. Eur Respir J.

[58] Liang W (2019). Non-invasive diagnosis of early-stage lung cancer using high-throughput targeted DNA methylation sequencing of circulating tumor DNA (ctDNA). Theranostics.

[59] Abbosh C (2023). Tracking early lung cancer metastatic dissemination in TRACERx using ctDNA. Nature.

[60] Seijo LM (2019). Biomarkers in Lung Cancer Screening: achievements, promises, and challenges. J Thorac Oncol.

[61] Li L, et al. Applying circulating tumor DNA methylation in the diagnosis of lung cancer. Precis Clin Med. 2019;2:45–56. 10.1093/pcmedi/pbz003 PMC8985769 35694699

[62] Bibikova M, et al. Liquid biopsy for early detection of lung cancer. Chin Med J Pulm Crit Care Med. 2023;01:200–6. 10.1016/j.pccm.2023.08.005 PMC11332910 39171286

