# Supplementary material for: Investigating causal associations between pneumonia and lung cancer using a bidirectional mendelian randomization framework
Source: BMC Cancer. 2024 Jun 11;24:721. doi: 10.1186/s12885-024-12147-3 (PMC11167773; doi:10.1186/s12885-024-12147-3)
Supplement: Supplementary file 1 — Supplementary Material 1 [file 12885_2024_12147_MOESM1_ESM.docx]

**Supplementary Figures**

**Figure S1. Scatter plots showing the causal association of pneumonia on lung cancer.**

**Figure S2. Leave-one-out plots for pneumonia on lung cancer.**

**Figure S3. Funnel plots for pneumonia on lung cancer.**

**Figure 1.** Scatter plots showing the causal association of pneumonia on lung cancer, with the slope of each line corresponding to estimated causal effect per method. **A,** pneumonia on LC, LUAD, LUSC, and SCLC (from left to right); **B,** bacterial pneumonia on LC, LUAD, LUSC, and SCLC (from left to right); **C,** viral pneumonia on LC, LUAD, LUSC, and SCLC (from left to right); **D,** asthma-related pneumonia on LC, LUAD, LUSC, and SCLC (from left to right); **E,** COVID-19 on LC, LUAD, LUSC, and SCLC (from left to right).

**Figure S2.** Leave-one-out plots for pneumonia on lung cancer. A, pneumonia on LC, LUAD, LUSC, and SCLC (from left to right); **B,** bacterial pneumonia on LC, LUAD, LUSC, and SCLC (from left to right); **C,** viral pneumonia on LC, LUAD, LUSC, and SCLC (from left to right); **D,** asthma-related pneumonia on LC, LUAD, LUSC, and SCLC (from left to right); **E,** COVID-19 on LC, LUAD, LUSC, and SCLC (from left to right).

**Figure S3.** Funnel plots for pneumonia on lung cancer. A, pneumonia on LC, LUAD, LUSC, and SCLC (from left to right); **B,** bacterial pneumonia on LC, LUAD, LUSC, and SCLC (from left to right); **C,** viral pneumonia on LC, LUAD, LUSC, and SCLC (from left to right); **D,** asthma-related pneumonia on LC, LUAD, LUSC, and SCLC (from left to right); **E,** COVID-19 on LC, LUAD, LUSC, and SCLC (from left to right).
